# *Pleurotus ostreatus* as a model mushroom in genetics, cell biology, and material sciences

**DOI:** 10.1007/s00253-024-13034-4

**Published:** 2024-02-19

**Authors:** Takehito Nakazawa, Moriyuki Kawauchi, Yuitsu Otsuka, Junxian Han, Daishiro Koshi, Kim Schiphof, Lucía Ramírez, Antonio G. Pisabarro, Yoichi Honda

**Affiliations:** 1 Graduate School of Agriculture, Kyoto University, Oiwake-Cho, Kitashirakawa, Sakyo-Ku, Kyoto, 606-8502 Japan; 2 Institute for Multidisciplinary Research in Applied Biology (IMAB), Public University of Navarra (UPNA), 31006 Pamplona, Spain

**Keywords:** Agaricomycete, Genome editing, Cell wall, Mycelial materials, Breeding, Wood degradation

## Abstract

**Abstract:**

*Pleurotus ostreatus*, also known as the oyster mushroom, is a popular edible mushroom cultivated worldwide. This review aims to survey recent progress in the molecular genetics of this fungus and demonstrate its potential as a model mushroom for future research. The development of modern molecular genetic techniques and genome sequencing technologies has resulted in breakthroughs in mushroom science. With efficient transformation protocols and multiple selection markers, a powerful toolbox, including techniques such as gene knockout and genome editing, has been developed, and numerous new findings are accumulating in *P. ostreatus*. These include molecular mechanisms of wood component degradation, sexual development, protein secretion systems, and cell wall structure. Furthermore, these techniques enable the identification of new horizons in enzymology, biochemistry, cell biology, and material science through protein engineering, fluorescence microscopy, and molecular breeding.

**Key points:**

*• Various genetic techniques are available in Pleurotus ostreatus.*

*• P. ostreatus can be used as an alternative model mushroom in genetic analyses.*

*• New frontiers in mushroom science are being developed using the fungus.*

## Introduction

Historically, the genetics of mushrooms have mostly been studied using two model mushrooms, *Coprinopsis cinerea* and *Schizophylum commune* (Raper and Miles 1958; Kües and Casselton 1992). These two species have relatively short life cycles, including sexual development and basidiospore formation, which can be completed in the laboratory. Many mutant strains have been isolated and characterized, and various basic properties of mushroom-forming fungi, such as mating type and fruiting body development, have been analyzed (Kamada 2002; Kües and Navarro-González 2015). These two mushrooms still have good potential as model mushrooms and offer opportunities to analyze new aspects of mushroom biology. Practically, popular cultivated mushrooms, such as *Lentinus edodes*, *Agaricus bisporus*,* P. ostreatus*,* P. eryngii*,* Flammulina velutipes*,* Agaricus marmoreus*,* Cyclocybe cylindracea*, and *Auriculalia heimer*, have been a target of breeding and a focus of interest in molecular analysis. Medicinal mushrooms, such as *Ganoderma lucidum* and *Cordyceps militaris*, have been used to analyze the biosynthetic pathways of terpenoids. However, many of them have slower growth rates, and it takes several weeks or even months to develop a fruiting body, which is essential for segregation analysis and conventional breeding with repeated crosses. Therefore, genetic properties were analyzed and deduced mostly using the conventional model mushrooms *C. cinerea* and *S. commune.*

During the last quarter-century, molecular genetic approaches have evolved into powerful tools for identifying, characterizing, and modifying genes with critical roles in a variety of physiological phenomena. These approaches include the use of molecular markers, transformations, gene targeting, RNA interference (RNAi), and genome editing. Using these techniques, it is possible to rapidly clone a gene responsible for a mutation in forward genetics and to demonstrate the function of a gene in reverse genetics using knockout, knockdown, and overexpression. It is possible to breed a new strain with modified regulation of the gene of interest. On the other hand, genome analysis has become very popular and sequence information of many filamentous fungi is available on the internet, mostly brought by the JGI 1000 fungal genome project (Varga et al. 2019; Miyauchi et al. 2020). Information technology using genome databases has made it easier to analyze individual species of cultivated mushrooms directly. It is possible to perform post-genomic analyses in non-cultivated wild mushrooms, including mycorrhiza, using the genome data of other fungi.

However, to utilize the benefits of combining modern molecular genetic tools and genome information technology, it is essential to have a practical and efficient transformation system and adequate genetic tool elements, such as selection markers. In *P. ostreatus*, these prerequisites have been well-established, and with easy cultivation and a relatively short life cycle, it is possible to carry out effective and rapid analyses using both forward and reverse genetic approaches. In this mini-review, we discuss recent progress in the molecular genetics of this fungus and demonstrate its status as a model mushroom in research topics such as wood degradation, sexual development, and cell wall structure. Future perspectives on cell biology and mushroom materials are also discussed.

## Genetic toolbox for *P. ostreatus*

In *P. ostreatus*, genetic markers were first characterized to analyze mating-type genes (Larraya et al. 1999a) and a linkage map was reported (Larraya et al. 2000). These were the starting points for molecular genetic research on this fungus, making it one of the best mushrooms equipped with various experimental techniques. The dikaryotic strain, N001, isolated from Navarra, Spain, and its monokaryotic derivatives, PC9 and PC15 (Larraya et al. 1999b), are distributed worldwide and are used as standard strains for molecular genetic analyses of this fungus. The first genome sequence analysis was performed on these strains (Riley et al. 2014) and recently updated using high-quality new-generation sequencing method (Lee et al. 2021).

### Genetic transformation system for *P. ostreatus*

Transformation of *P. ostreatus* was first reported by Peng et al. (1992). They described development of a transformation system employing a recombinant plasmid harboring a selectable resistance marker to hygromycin B and recovery of rearranged plasmids from the transformants. Furthermore, the same group reported possible extrachromosomal maintenance of unintentionally isolated recombinant plasmids containing a DNA sequence from bacteriophage P1 (Herzog et al. 1995). In 1996, a recombinant drug-resistant marker to bialaphos was developed and successfully used for transformation (Yanai et al. 1996). Despite their prominent achievements as frontier innovators, these transformation techniques are difficult to reproduce and have not become popular in other laboratories, most likely because of their low efficiency and sensitive protoplast yields, depending on the strain and culture conditions.

A practical and stable transformation system for *P. ostreatus* was first developed when a novel drug-resistance marker of the systemic fungicide carboxin was developed (Honda et al. 2000). A carboxin-resistance marker was constructed to introduce one amino acid change in a gene encoding the iron-sulfur protein (Ip) subunit of mitochondrial succinate dehydrogenase complex II, which is the target site of a widely used agricultural chemical to control corn-smut pathogens. Being a homologous marker gene, the carboxin resistance gene offers self-cloning and reliable expression when introduced into cells. Moreover, it is dominant and background colonies were seldom observed during the screening. This situation has allowed the optimization of transformation protocols in *P. ostreatus* and the isolation of a *ku80* deletant (Salame et al. 2012) from the monokaryon PC9 (Larraya et al. 1999b), which established a high-frequency gene targeting system by homologous recombination in the absence of a non-homologous end joining (NHEJ) DNA repair system. Using the *ku80* deletant, Nakazawa et al. (2016) isolated disruptants of *pyrG*, whose disruption conferred 5-fluorotinic acid (5-FOA) resistance and demonstrated *pyrG*-complementary transformation. They developed marker recycling methods for multiple gene disruptions using the counter-selection of *pyrG* and *fcy1*, disruption of which confers 5-fluorocytocine (5-FC) resistance. Moreover, a drug-resistance marker for nourseothricin has been developed (Matsunaga et al. 2017). These efforts provide a platform for *P. ostreatus* as one of the most useful agaricomycetes for molecular genetic studies.

Recently, unstable drug-resistant transformants have been reported in *Gelatoporia* (formerly *Ceriporiopsis*) *subvermispora*, depending on the introduction of a recombinant plasmid harboring a hygromycin-resistance marker (Honda et al. 2019). This result strongly suggests transient and possibly extrachromosomal expression of the marker gene. This plasmid lacks the DNA sequence from the P1 phage reported by Herzog et al. (1995). It is plausible that similar transient transformations can occur in many other transformation systems, depending on the balance between screening pressure and the expression level of the selection marker gene. Additionally, the mode of action of the marker gene is important for detecting transient transformation. The hygromycin resistance marker encodes a hygromycin phosphotransferase, which inactivates the drug. It can confer different levels of drug resistance depending on its expression levels, allowing transient drug-resistant transformants to be selected under suitable screening conditions. Using the clustered regularly interspaced short palindromic repeats (CRISPR)/CRISPR-associated protein 9 (Cas9) genome editing system in *P. ostreatus*, Koshi et al. (2022, also see below) demonstrated that a plasmid expressing Cas9 and guide RNA (gRNA) allows successful genome editing through transient expression of these genes as well as hygromycin resistance markers used for temporal screening of the transformant. In the future, the transient expression of introduced genes could be used for various objectives, such as marker recycling. However, the integration of foreign DNA fragments at random sites on chromosomes can be a potential problem, as in the case of stable transformations.

### CRISPR/Cas9 technologies in mushrooms

CRISPR/Cas9 is an adaptive immune system found in archaea and bacteria (Song et al. 2019) that has recently been utilized as a versatile gene-targeting tool. CRISPR/Cas9 is composed of two main parts: a Cas9 endonuclease for generating double-stranded DNA breaks (DSBs) and a single guide RNA (sgRNA) to recruit Cas9 to the target site on the chromosome through the formation of hybrid duplex nucleotides. After targeting DSBs, errors sometimes occur during the NHEJ repair process, introducing mutations at the target site. CRISPR/Cas9-assisted gene targeting protocols are divided into two major strategic categories: DNA-based and pre-assembled Cas9 ribonucleoprotein (RNP)-based methods. The former introduces foreign DNA, such as a recombinant plasmid containing expression cassettes for both Cas9 and sgRNA. The latter introduces the Cas9-sgRNA RNP complex, which was prepared or purchased separately, followed by a pre-assembly step in vitro.

Unlike conventional gene targeting using homologous recombination, CRISPR/Cas9 does not require NHEJ-deficient host strains, such as *ku* or *lig4* deletants, which are isolated only in a limited number of mushroom species, such as *S. commune* (De Jong et al. 2010), *C. cinerea* (Nakazawa et al. 2011), and *P. ostreatus* (Salame et al. 2012). Thus, this method can be applied to other agaricomycetes using a practical genetic transformation system. Recently, the DNA-mediated CRISPR/Cas9 was reported in agaricomycetes, such as *C. cinerea* (Sugano et al. 2017), *G. lingzhi* (Qin et al. 2017),* G. lucidum* (Qin et al. 2017; Liu et al. 2020), *P. ostreatus* (Boontawon et al. 2021a; Xu et al. 2022; Yamasaki et al. 2022; Koshi et al. 2022), *P. eryngii* (Wang et al. 2021), *L. edodes* (Moon et al. 2021; Kamiya et al. 2023), *Flammulina filiformis* (Liu et al. 2022a), *G. subvermispora* (Nakazawa et al. 2022), and *Agaricus bisporus* (Choi et al. 2023) (Table 1). In addition to the expected small insertion or deletion mutations at the target site, integration of introduced DNA fragments has been frequently observed in *P. osteatus* (Yamasaki et al. 2022; Koshi et al. 2022). This could be a result of high selection pressure for hygromycin resistance (Yamasaki et al. 2022; Koshi et al. 2022) but similar phenomena may occur in many other cases. However, it is difficult to detect these mutants in genomic PCR experiments, and such an amplification failure could eliminate these isolates from subsequent nucleotide sequence determination.

**Table 1:** List of agaricomycetes for which CRISPR/Cas9 have been reported

Species	Type of the protocol	References
*Agaricus bisporus*	RNP-based	Choi et al. (2023)
*Coprinopsis cinerea*	DNA-based	Sugano et al. (2017)
	RNP-based	Pareek et al. (2022)
*Dichomitus squalens*	RNP-based	Kowalczyk et al. (2021)
*Flammulina filiformis*	DNA-based	Liu et al. (2022a)
	RNP-based	Liu et al. (2022b)
*Ganoderma lingzhi*	DNA-based	Qin et al. (2017)
*Ganoderma lucidum*	DNA-based	Qin et al. (2017)
		Liu et al. (2020)
		Tu et al. (2021)
	RNP-based	Eom et al. (2023)
*Gelatoporia subvermispora*	DNA-based	Nakazawa et al. (2022)
*Lentinula edodes*	DNA-based	Moon et al. (2021)
		Kamiya et al. (2023)
*Pleurotus eryngii*	DNA-based	Wang et al. (2021)
*Pleurotus ostreatus*	DNA-based	Boontawon et al. (2021a)
	RNP-based	Boontawon et al. (2021b; 2023)

The RNP-based method has been reported for some agaricomycetes, such as *S. commune* (Jan Vonk et al. 2019), *P. ostreatus* (Boontawon et al. 2021b), *Dichomitus squalens* (Kowalczyk et al. 2021), *F. filiformis* (Liu et al. 2022b), *C. cinerea* (Pareek et al. 2022), and *G. lucidum* (Eom et al. 2023) (Table 1). Technically, this protocol can be applied to species lacking a suitable selection marker gene, provided that protoplasts can be generated efficiently. It does not require laborious steps, such as selecting appropriate promoters for Cas9 and gRNA expression or codon optimization of Cas9 for each fungal species. However, at present, the efficiency of genome editing using RNP-based methods may be lower than that of DNA-based methods (Boontawon et al. 2021b). The major challenge of the RNP-based method is the lack of an efficient selection system; this method has been applied only to a special target gene whose disruption makes it possible to screen for, such as *pyrG* (Boontawon et al. 2021b; Kowalczyk et al. 2021; Eom et al. 2023) or *fcy1* (Boontawon et al. 2023), unless a foreign selection marker was also introduced (Jan Vonk et al. 2019; Boontawon et al. 2021b; Kowalczyk et al. 2021; Pareek et al. 2022). To overcome this problem, RNP-dependent CRISPR/Cas9 with two target genes, one for screening and the other the gene of interest, was developed in *P. ostreatus* (Boontawon et al. 2023).

CRISPR/Cas9 can target the disruption of multiple genes in a single operation using multiple gRNAs. Using a polycistronic tRNA and CRISPR guide RNA approach, triple-gene disruption via DNA-dependent CRISPR/Cas9 was demonstrated in *P. ostreatus* and for the first time in agaricomycetes (Xu et al. 2022). Generally, the number of genes that can be disrupted by homologous recombination in conventional gene targeting is limited by the availability of different selection markers. This causes serious problems when multiple genes are disrupted. Multiple gene disruption with CRISPR/Cas9 may overcome this problem. For example, by creating a sextuple disruption strain for lignin-modifying enzyme (LME)-encoding genes, the crucial role of LMEs in lignin degradation by *P. ostreatus* was demonstrated (Nakazawa et al. 2023b; see below).

CRISPR/Cas9 is a promising technique for rapid and efficient improvement of crops. In some countries, including the USA, Japan, and Australia, improved crops without transgenes or foreign DNA are excluded from genetically modified organism regulations (Tsuda et al. 2019; Entine et al. 2021). With DNA-dependent CRISPR/Cas9, marker-free genome editing in *P. ostreatus* using transient transformation of the plasmid harboring Cas9 and gRNA was reported (Koshi et al. 2022). In this study, plasmid fragments were frequently integrated at the target site, probably due to high NHEJ activity, making it laborious to screen for transient transformants free of plasmid insertion. Optimization of the screening conditions may increase the efficiency of transient and non-chromosome-integrated transformations, which may lead to the isolation of foreign DNA-free genome-edited strains.

RNP-based methods can introduce mutations at the target site in a foreign DNA-independent manner, which may be beneficial for isolating strains free of heterogeneous DNA sequences. However, in *G. lucidum*, frequent integration of contaminated and mitochondrial DNA fragments at the target site has been observed in RNP-dependent genome editing (Eom et al. 2023). Integration of unexpected DNA sequences at the target and off-target sites could be a fatal problem in CRISPR/Cas9, both in DNA- and RNP-dependent protocols, although successful isolation of small insertion/deletion mutations has been reported in *P. ostreatus* (Boontawon et al. 2023).

Moreover, we are currently developing a new methodology for foreign DNA-free genome editing with CRISPR/Cas9 using the dikaryotic state. By supplying Cas9 and sgRNA from one nucleus as a donor, the intended genome editing was successfully carried out in the other recipient nucleus, followed by dedikaryozation through protoplast regeneration (Koshi et al. to be published elsewhere). However, with any CRISPR/Cas9 protocol, checking the genome sequence is necessary to exclude unintended rearrangements or integration of foreign DNA. The accumulated knowledge of CRISPR/Cas9 in *P. ostreatus* provides a foothold for expanding gene-targeting tools and will contribute to research on safer and more acceptable molecular breeding techniques in other agaricomycetes.

## Wood degradation

Some agaricomycetes, called “wood-rot fungi,” act as key decomposers in forest ecosystems by degrading the complex lignocellulose matrix. Elucidation of the mechanisms underlying degradation of lignin, a recalcitrant heteropolymer, by these fungi, especially by “white-rot fungus,” would contribute to the development of new methods for efficient and ecofriendly utilization of lignocellulose as a sustainable resource. Lignin degradation mechanisms can also be utilized to remove persistent organic pollutants from the environment (Mori et al. 2021).

Early progress in elucidating wood degradation mechanisms was achieved using the white-rot fungus *Phanerochaete chrysosporium*. Various enzymes that may play important roles in the degradation of cellulose, hemicellulose, and lignin have been characterized, and the genes encoding these enzymes have been cloned. Following this, a special kind of LME, the versatile peroxidase (VP), was isolated and characterized in *P. ostreatus* (Kamitsuji et al. 2004) and *P. eryngii* (Martínez et al. 1996), and recombinant enzymes produced using homologous (Tsukihara et al. 2006) and bacterial expression systems (Fernández-Fueyo et al. 2014) were characterized to demonstrate the structure and functional relationship of these enzymes, respectively. Since genome analysis has become popular in filamentous fungi through the JGI 1000 fungal genome project, an increasing number of wood-rot fungi have been used to analyze wood degradation mechanisms. It is possible to carry out transcriptomic, proteomic, and metabolomic analyses using various fungi, which reveal the common and unique features of these species. Substantial efforts have been devoted to elucidating the mechanisms of wood degradation over the years using various approaches (Fernandez-Fueyo et al. 2012; Zhang et al. 2019; Kijpornyongpan et al. 2022).

However, low accessibility to molecular genetic approaches, especially gene targeting, has prevented the elucidation of which mechanisms and factors are actually required for the biodegradation of natural wood substrates, as it is difficult to examine the precise effects of the loss of each mechanism or factor. *P. ostreatus* is a unique white-rot fungus for which many useful molecular genetic tools, including efficient gene targeting, are available (Honda et al. 2000; Salame et al. 2012; Nakazawa et al. 2016; Matsunaga et al. 2017), making it a valuable model for studying lignocellulose degradation using a molecular genetics approach. Here, we review recent studies investigating the mechanisms underlying lignin degradation by *P. ostreatus*.

### Genes for degrading enzymes

Nine lignin-modifying peroxidase-encoding genes have been predicted in the genome of *P. ostreatus* (Knop et al. 2015): three were predicted to be versatile peroxidases (VPs) and six were predicted to be manganese peroxidases (MnPs) (Fernández-Fueyo et al. 2014). These white-rot fungi-specific peroxidases are considered key players in lignin degradation because they can oxidize model lignin compounds and depolymerize synthetic lignin, including the dehydrogenase polymer lignin, in vitro (Kondo et al. 1990; Eggert et al. 1996). However, the natural lignin in plant cell walls is distinct from synthetic molecules; for example, natural lignin is a complex water-insoluble heteropolymer. To demonstrate that these enzymes play an important role in natural lignin degradation, single-gene deletions generated by homologous recombination were analyzed. Salame et al. (2014) showed that *vp2* deletion slightly reduces the lignocellulose-degrading ability of cotton stalks, suggesting that VP2 is dispensable for lignin degradation by *P. ostreatus*, probably because of the redundancy between VPs and MnPs (Salame et al. 2013). Recently, CRISPR/Cas9 has been used to generate multiple gene disruptants of *mnp* and *vp*. Additionally, one laccase-encoding gene, *lac2*, was disrupted because its transcript accumulation was high when grown on beech wood sawdust medium supplemented with wheat bran (BWS; Wu et al. 2020). Lignin-degrading abilities were mostly lost in the *vp2*/*vp3*/*mnp3*/*mnp6*/*mnp2/lac2* sextuple-gene disruptants but not in the *vp2*/*vp3*/*mnp3*/*mnp6*/*mnp2* and *vp2*/*vp3*/*mnp3*/*mnp6*/*lac2* quintuple-gene disruptants grown on BWS, clearly showing their essential role (Nakazawa et al. 2023b). This is the first experimental evidence indicating that these LMEs play essential roles in wood lignin biodegradation by white-rot fungi.

### Genes for regulators of lignocellulose degradation

The availability of a reverse genetic approach based on high-frequency gene targeting and a forward genetic approach using genome sequence information have allowed for the identification of various regulators possibly involved in the transcriptional expression of lignocellulose-degrading enzymes in *P. ostreatus* (Table 2). For example, Wtr1 and Gat1, agaricomycete-specific Zn_2_Cys_6_ and GATA DNA-binding transcription factors, respectively, were identified by forward genetic strategies using randomly mutagenized strains (Nakazawa et al. 2017a; 2019). Single gene mutations in *wtr1* and *gat1*, isolated in gene knockout experiments, resulted in defects in the decolorization of the dyes Orange II and Remazol Brilliant Blue R, which are used to examine ligninolytic activity in *P. ostreatus* on agar plates. They are possible positive regulators of lignin degradation because the lignin-degrading ability in BWS is reduced in single-gene mutants or deletions of these genes. Yoav et al. (2018) analyzed *P. ostreatus cre1* deletants and overexpressing strains. Cre1 is a DNA-binding transcription factor homologous to *Aspergillus nidulans* CreA, which is an important carbon catabolite repressor. Overexpression and deletion of *P. ostreatus cre1* resulted in reduced and enhanced lignocellulose-degrading ability of *P. ostreatus*, respectively (Yoav et al. 2018). In these mutants, the expression levels of many lignocellulose-degrading enzymes (encoding genes) on lignocellulose substrates are greatly altered (Wu et al. 2020, 2021a; Yoav et al. 2018). Alfaro et al. (2020) demonstrated that glucose counteracts the wood-dependent induction of cellulolytic enzymes but not lignin degrading peroxidases. It is of interest whether Cre1 and glucose may contribute to the regulation of these genes. Transcriptional changes have also been observed in single-gene mutants of two putative chromatin remodeler-encoding genes, *hir1* and *chd1*, in *P. ostreatus* (Nakazawa et al. 2017a, b; Wu et al. 2021b). Based on these findings, we isolated single-gene deletions related to histone modifications. Altered expression patterns were observed for some gene deletions (Okuda et al. 2021, unpublished). Future studies are required to investigate how each regulator alters transcriptional expression, which will provide a methodology for the artificial induction and regulation of ligninolytic, cellulolytic, and hemicellulolytic systems.

**Table 2:** List of genes possibly involved in transcriptional regulation of putative lignocellulose-degrading enzymes in *P. ostreatus* that have been identified to date

Gene	Protein encoded	Phenotype	Reference
*chd1*	A putative chromatin remodeler	Deletants^2^: decrease in lignin loss after culturing on BWS	Nakazawa et al. (2017a)
*cam1*	Calmodulin-like protein	KD^2^ and OE^2^: increase and decrease in MnP activity, respectively	Suetomi et al. (2015)
*ccl1*	A component of histone H3 methylation at lysine 4	Deletants^2^: transcriptional upregulation of putative five GH6-, GH7-, and AA9-encoding genes on BWS	Okuda et al. (2021)
*cre1*	A putative C2H2-type DNA-binding transcription factor	Deletants^2^ and OE^2^: increase and decrease in lignocellulose loss after culturing on wheat straw, respectively	Yoav et al. (2018)
*gat1*	A GATA-type DNA-binding transcription factor^1^	Deletants^2^: decrease in lignin loss after culturing on BWS	Nakazawa et al. (2019)
*hir1*	A putative chromatin remodeler	Deletants^2^: decrease in lignin loss after culturing on BWS	Wu et al. (2021b)
*mpk1b*	A putative MAP kinase	OE^2^: transcriptional upregulation of putative three GH6-, GH7-, and AA9-encoding genes on BWS	Okuda et al. (2021)
*ssp1*	Unknown	KD^2^ and OE^2^: decrease and increase in Aryl-alcohol oxidase activity, respectively	Feldman et al. (2017, 2019)
*wtr1*	A Zn_2_Cys_6_-type DNA-binding transcription factor^1^	Deletants^2^: decrease in lignin loss after cultivation on BWS	Nakazawa et al. (2017a)

^1^DNA-binding capacity to specific nucleotide sequences has been analyzed (unpublished)

^2^ “Deletants” indicates gene deletants generated using homologous recombination; “KD” indicates knock down using RNA interference; “OE” indicates over expression

### Expanding molecular genetics analyses in other wood-decaying fungi

Functional analysis of the *G. subvermispora* gene homologous to *P. ostreatus gat1* was conducted (Table 2) using DNA-based CRISPR/Cas9. Similar to *P. ostreatus gat1* mutants, lignin-degrading abilities were mostly lost in *G. subvermispora gat1* disruptants grown on BWS (Nakazawa et al. 2023a). This suggests that Gat1 is a conserved regulator of the ligninolytic system in white-rot fungi. On the other hand, *pex1* gene (peroxisomes) is essential for lignin degradation in *P. ostreatus*, but not in *G. subvermispora* (Nakazawa et al. 2017b, 2023a). Comparative genomic, transcriptomic, and secretome studies have proposed theories on the diversity of mechanisms underlying lignin (or lignocellulose) degradation in white-rot fungi (Fernandez-Fueyo et al. 2012; Hori et al. 2014). However, these theories have not yet been experimentally examined. Gene targeting experiments in various white-rot fungi would be useful for this purpose. The accumulation of knowledge from “comparative molecular genetics” will reveal the dynamics of these mechanisms during the evolution of white-rot fungi.

### Protein secretion pathway

**Fig. 1:**
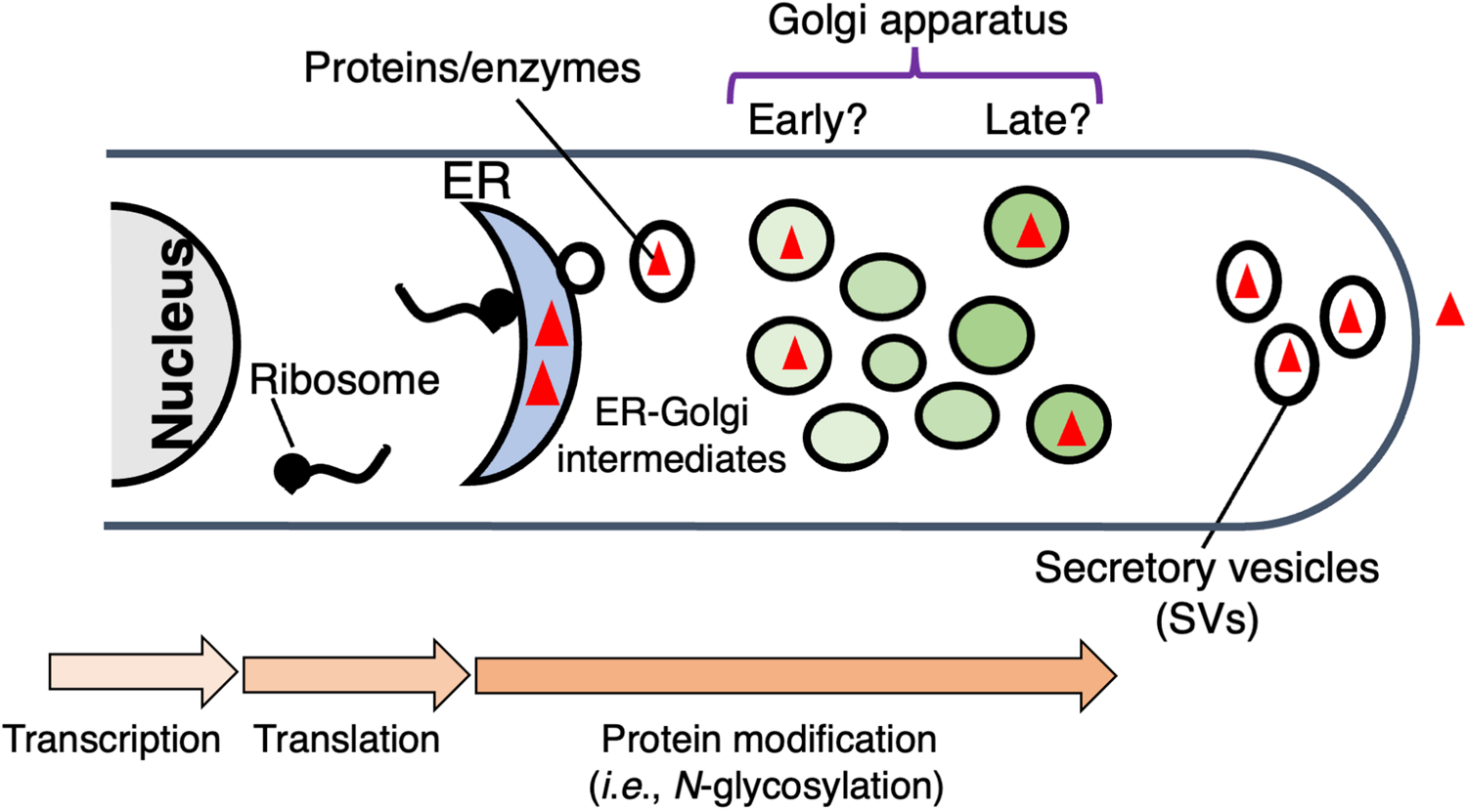
Proposed secretion pathway of proteins in *P. ostreatus*. This schematic presentation was drawn based on previous studies in Ascomycetes (Shoji et al. 2008) and our recent study that visualized organelles with recombinant fluorescent proteins in *P. ostreatus* (Kurebayashi et al. 2023)

The production of lignocellulose-degrading enzymes can be regulated or affected at post-transcriptional or translational steps. For examples, changes at the transcription level were not consistent with those in enzyme activities of LMEs in some white-rot fungi, such as *Phanerochaete chrysosporium* and *Trametes versicolor* (Sakamoto et al. 2010; Přenosilová et al. 2012). One possible mechanism responsible for such inconsistencies may be protein secretion, protein folding, modification, and trafficking (Fig. 1). Investigating the mechanisms or pathways of protein secretion as well as transcriptional regulation may contribute to the enhancement and modulation of the production level of LMEs (and lignin-degrading abilities) by white-rot fungi. However, protein secretion pathways in white-rot fungi have not yet been well studied. Generally, secretory proteins are transported outside of cells through the endoplasmic reticulum, Golgi apparatus, and secretory vesicles in eukaryotes (Shoji et al. 2008) (Fig. 1). Considering that these organelles have hardly been studied in *Agaricomycetes*, future studies are needed to confirm whether LMEs are also secreted via similar secretion pathways to investigate the possible regulatory mechanisms and/or bottlenecks in their production. Recently, organelles participating in protein secretion were successfully visualized using recombinant fluorescent proteins in *P. ostreatus*, suggesting differences in the Golgi apparatus between *P. ostreatus* and ascomycetes (Kurebayashi et al. 2023). These fluorescence microscopy techniques for *P. ostreatus* are expected to provide new insights into the cell biology of agaricomycetes.

## Sexual development

As one of the most popular and commercially important edible mushrooms worldwide, breeding of *P. ostreatus* is important. The accelerated development of powerful methodologies for gene targeting, such as CRISPR/Cas9, has allowed molecular breeding to quickly improve the cultivation and food traits of *P. ostreatus* (Yamasaki et al. 2022) without laborious and time-consuming crossing steps.

### Dikayrosis

Fruiting bodies are generally produced from dikaryotic strains, which are formed when two monokaryotic strains with compatible alleles at both *A* and *B* loci are fused (Kamada 2002). Therefore, dikaryosis or dikaryon formation is important in the sexual life cycle and is exclusively observed in basidiomycetes. The underlying mechanisms have been investigated in the model tetrapolar agaricomycetes *C. cinerea* and *S. commune* (Kamada 2002; Kües and Navarro-González 2015; Raudaskoski 2015). *P. ostreatus* is also tetrapolar and is evolutionarily close to these model species (Riley et al. 2014). Moreover, our microscopic observations did not reveal any differences in clamp cell morphogenesis or distribution of nuclei in dikaryotic hyphae between *P. ostreatus* and the conventional model species (Boontawon et al. 2021c; unpublished). However, recent functional analyses of *P. ostreatus* genes homologous to *C. cinerea pcc1* and *clp1* (Murata et al. 1998; Inada et al. 2001) suggested some differences in the *A-* and *B*-regulated pathways and/or roles of Pcc1 between *C. cinerea* and *P. ostreatus* (Boontawon et al. 2021c): like *C. cinerea*, monokaryotic *pcc1* disruptants formed incomplete fruiting bodies without mating, but unlike *C. cinerea*, no pseudo-clamp cells were observed in their hyphae. At present, it is difficult to further investigate the mechanisms underlying dikaryosis and conduct functional analyses of *pcc1* and *clp1* in *P. ostreatus* because monokaryotic strains in which *A-* and/or *B-*regulated pathways are activated are not available, unlike in *C. cinerea* and *S. commune* (Koltin 1970; Kües 2015). Future studies are needed to generate such *P. ostreatus* strains, which will enable us to conduct detailed molecular genetic studies on dikaryosis as well as generate monokaryotic or homokaryotic fruiting strains. These strains will be useful for efficient and rapid molecular breeding.

### Fruiting body development

Many genes affecting fruiting initiation and development have been identified in *C. cinerea* and *S. commune* (Kamada 2002; Ohm et al. 2011). However, the environmental factors and conditions (i.e., nutrients, temperature, and light) that trigger fruiting in *P. ostreatus* are different from those of model agaricomycetes. For example, a temperature downshift (to 4–18 °C from 25–30 °C) is essential for *P. ostreatus*, but not *C. cinerea* in most cases. Heat shock induces fruiting in *S. commune* (Ohm et al. 2012). Blue light is required for both fruiting initiation and maturation (stipe elongation, pileus expansion, and meiosis in basidia) in *C. cinerea* (Kües and Navarro-González 2015; Liu et al. 2022a, b, c) but only for initiation in *P. ostreatus*. In addition, the shape of the fruiting bodies of *P. ostreatus* is distinct from that of many other agaricomycetes. Therefore, there are many differences in the mechanisms underlying fruiting initiation and morphology of *P. ostreatus* compared to *C. cinerea* and *S. commune*.

In this context, we analyzed the effects of *exp1* or *eln3* deletion in *P. ostreatus*. *exp1* is essential for pileus expansion and *eln3* for stipe elongation (expansion of stipe cells) in *C. cinerea* (Arima et al. 2004; Muraguchi et al. 2008). However, no obvious mutant phenotypes were observed in fruiting bodies formed from dikaryotic *P. ostreatus eln3* or *exp1* deletants (unpublished data). In *C. cinerea*, *eln3* encodes a putative glycosyltransferase of unknown function, and another putative glycosyltransferase-encoding gene is essential for the expansion of stipe cells (Kamada and Muraguchi, personal communication). Furthermore, biochemical studies have suggested the involvement of cell-wall polysaccharide, chitin, and glucan remodeling in stipe cell expansion (Kamada et al. 1991; Kang et al. 2019; Zhou et al. 2019). In *P. ostreatus*, our microscopic observations revealed elongated stipe cells; however, unlike in *C. cinerea* (Kamada and Takemaru 1977), stipe elongation does not seem to be triggered at a specific time, but gradually occurs in *P. ostreatus*. Therefore, it is unclear whether the difference in the effects of *eln3* mutations reflects the remodeling program or the fundamental structures of cell wall polysaccharides between the two agaricomycetes.

Efficient methodologies for molecular breeding should also be developed for quick application. However, even though efficient gene-targeting is available for *P. ostreatus* today, it is still laborious and time-consuming to analyze the precise effects of the deletion/disruption of a certain gene on fruiting development. This is because dikaryotic strains, in which a target gene is deleted/disrupted in both nuclei, should be generated (Nakazawa et al. 2017b; Okuda et al. 2021). Recently, gene targeting in both nuclei of a dikaryon in a single transformation procedure with plasmid-based CRISPR/Cas9 was reported (Yamasaki et al. 2022), which would allow for quick and efficient molecular breeding.

### Spore formation

One of the important and long-standing breeding targets for *P. ostreatus* is a “sporeless” phenotype (Eger et al. 1976). This is because *P. ostreatus* produces an enormous amount of basidiospores, and their dispersal in mushroom cultivation facilities causes allergic reactions in employees as well as facility disruption. Basidiospores are sexual spores formed outside the basidium, where meiosis occurs. The core meiotic program has been conserved among fungi for over half a billion years of evolution, including in *Saccharomyces cerevisiae* and *C. cinerea* (Burns et al. 2010). Therefore, genes that play an important role in meiotic progression are promising targets for molecular breeding of sporeless strains (Lu et al. 2003). In fact, the meiosis-related genes, *msh4* and *mer3*, have been shown to be responsible for sporeless phenotypes in *C. cinerea*, *P. ostreatus*, and *P. pulmonarius* (Namekawa et al. 2005; Okuda et al. 2013; Lavrijssen et al. 2020; Yamasaki et al. 2021, 2022).

However, *P. ostreatus msh4* or *mer3* deletion/disruption does not always impair basidiospore production completely (Yamasaki et al. 2021). The *P. pulmonarius msh4* mutant also produces basidiospores (Okuda et al. 2013). Furthermore, Cummings et al. (1999) reported that sporeless phenotypes and hygromycin-B resistance do not co-segregate among F_1_ progeny from most *C. cinerea* mutants isolated after restriction enzyme-mediated integration. It seems that meiosis is an important, but not essential, step in spore formation. In *C. cinerea*, both post-meiotic and meiotic mutants have been isolated as sporeless mutants (Lu et al. 2003), suggesting that the post-meiotic process may be an alternative target. We are currently identifying the genes essential for this process in *P. ostreatus*. To date, two genes have been identified. These genes may be promising targets for the universal breeding of sporeless strains, as mutations in these genes completely impair basidiospore production, independent of the genetic background (unpublished).

## Cell wall structure and mushroom materials

Wood-rot fungi have multipurpose potential as they can create renewable materials from mycelia that can be grown on lignocellulosic biomass as a substrate. Mycelial materials have potential applications in packaging, leather alternatives, elastomers, and many other materials (Jeong et al. 2023). Despite their potential, it is challenging to produce mycelia that are strong, flexible, and durable enough to be viable alternatives to materials such as animals and artificial leathers. Mushroom cell walls play important roles in hyphal morphogenesis, mechanical strength, and interactions with the environment, and are therefore of interest for the development of mushroom materials as well as for applications in medicine and agriculture (Jeong et al. 2023; Raman et al. 2022). *P. ostreatus* is a promising species for research and is one of the most mentioned and published fungal species in the field of mycelium-based composites (Sydor et al. 2022).

Glucans, the major cell wall components in mushrooms, also have great potential in medicine and industry as they have anticarcinogenic, immune-stimulating, and antioxidant properties (Nagy et al. 2023). The β-glucan from *P. ostreatu*s is known as pleuran, and its structure and biological properties have been previously studied (Ohno et al. 2012). Pleuran is a polysaccharide with a molecular weight of 600–700 kDa comprising a β-1,3-linked main chain and β-1,6-linked branches of every four saccharides (Ohno et al. 2012). Pleuran, like other basidiomycete-derived β-glucans, also has immunostimulatory effects (Ohno et al. 2012). Therefore, in addition to improving the strength of biomaterial production, β-glucan modification has the potential to improve medical activity.

Chitin is another key cell wall component in mushrooms and is a raw material with many industrial applications. Chitin is thought to affect mycelial strength and cell wall rigidity; hence, it is important to produce stronger and more desirable mushroom materials (Raman et al. 2022). Furthermore, most industrial chitin is sourced unsustainably from seafood byproducts; however, fungi-sourced chitin has recently gained attention as a less wasteful, allergen-free, vegan, and sustainable chitin source (Abo Elsoud and El Kady 2019; Jones et al. 2020). Cell-wall gene engineering has the potential to improve chitin production in fungi.

To develop more sustainable, affordable, and accessible mushroom-derived materials, it is important to understand the cell wall structures of basidiomycetes. *P. ostreatus* is a good model for investigating the cell wall structure, genes involved in cell wall synthesis in mushrooms, and mycelial material production.

### Cell wall structure

Fungal cell walls perform diverse functions in maintaining cell integrity and rigidity (Alimi et al. 2023). Because of their large range of critical roles, the cell wall components of fungi often make up a large part of the body weight (up to 50%), and approximately one-sixth of the genes in the fungal genome are thought to be involved in cell wall synthesis (Alimi et al. 2023).

In filamentous fungi, the main structural components of the cell wall of vegetative mycelia are β-1,3-glucan, chitin, and α-1,3-glucan, to which proteins and other polysaccharides are attached (Gow et al. 2017; Ruiz-Herrera and Ortiz-Castellanos 2019). In *P. ostreatus*, the cell wall components of vegetative mycelia were analyzed using the alkaline fractionation method, and it was confirmed that the most abundant cell wall polysaccharide is β-glucan, followed by chitin, and α-glucan was observed as a small portion (Kawauchi et al., to be published elsewhere). Interestingly, in *P. ostreatus*, lower amounts of α-glucan but higher amounts of β-glucan were found than in the ascomycete *Aspergillus* (Yoshimi et al. 2022).

**Fig. 2:**
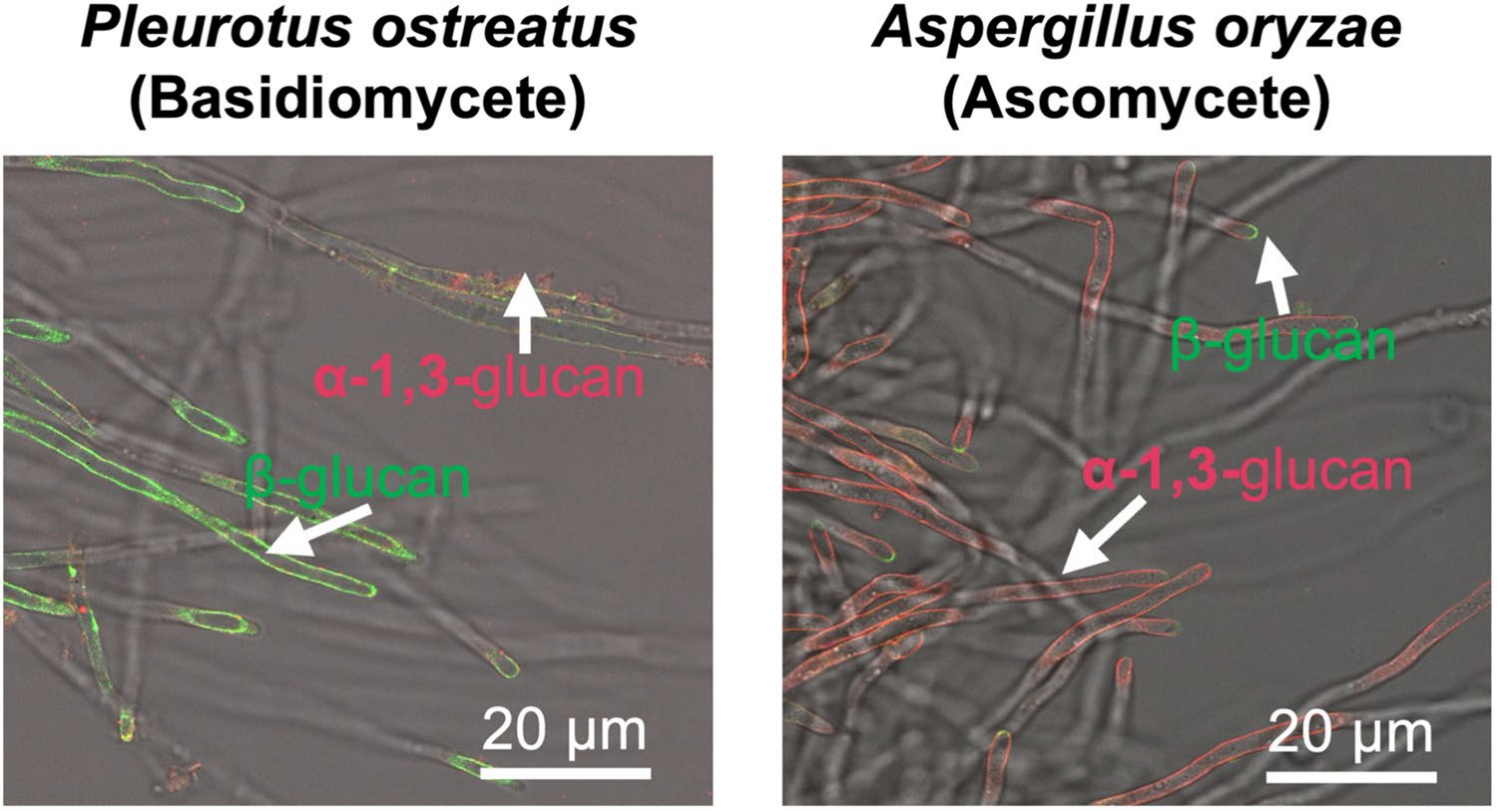
Comparison of cell wall surface glucans localization between the basidiomycete *P. ostreatus* and the ascomycete *A. oryzae*. For this comparison, *P. ostreatus* 20b (Salame et al. 2012) and *A. oryzae* NS4 Δ*ligD* (Mizutani et al. 2008) strains were cultivated in liquid culture. The obtained mycelia were stained with DCD-tetraRFP (α-1,3-glucan binding red fluorescent protein) and BGBD-GFP (β-glucan binding green fluorescent protein) and then observed under a confocal microscope. In this figure, β-glucan and α-1,3-glucan are depicted as green and red, respectively. Scale bar: 20 μm

Details of the cell wall structure of vegetative mycelia have been investigated often in pathogenic ascomycete fungi and to a much smaller extent in basidiomycetes; this is likely due to a range of factors, including the complexity and diversity of cell wall-related genes, lack of model systems, or difficulty in genetic manipulation (Nagy et al. 2023). In agaricomycetes, pioneering work was done by Wessels and colleagues to analyze cell wall structure based on alkaline fractionation methods using the mycelia of *S. commune* (Bartnicki-García 1999). Alkaline fractionation has long been used to analyze cell wall structures by isolating polysaccharides based on solubility differences in their constituent monosaccharides and bonding modes (Fontaine et al. 2000). The isolated polysaccharides can then be identified using several methods, such as enzymatic treatment, methylation analysis, X-ray spectroscopy, and nuclear magnetic resonance (NMR). Through analysis conducted by Wessels and colleagues, the outermost layer of the vegetative mycelium of *S. commune* was determined to be a β-glucan layer containing β-1,3 and β-1,6 linkages. Furthermore, solid NMR has also been used to analyze the cell wall in *S. commune* (Ehren et al. 2020), and the results suggest large structural differences compared to previous studies with the same analysis in the ascomycete *Aspergillus fumigatus* (Ehren et al. 2020; Kang et al. 2018). In this analysis, the cell wall of the ascomycete *A. fumigatus* was reported to contain α-1,3-glucan in the outer layer and β-glucan in the inner layer (Kang et al. 2018). On the other hand, the cell wall of *S. commune* has been found to have β-glucan in the outer layer and α-1,3-glucan in the inner layer (Ehren et al. 2020). Recently, Otsuka et al. developed a fluorescent probe for cell wall polysaccharide visualization (Otsuka et al. 2022a; b). The α-1,3-glucan probe (DCD-tetraRFP) and the β-glucan probe (BGBD-GFP) are fusion proteins of the substrate binding domain of hydrolytic enzymes (AGBD from *Bacillus circulans* and BGBD from *Lisobacter enzymogenes*) and a fluorescent protein, allowing this method to easily label polysaccharides in living mycelia of fungi. A chitin-binding fluorescent protein (ChBD from *B. circulans* fused with GFP) has also been reported to label the mycelial chitin of *S. commune* (Yano et al. 2011). Using these probes and cell wall dyes, Otsuka et al. successfully labeled and visualized the cell wall structure of the basidiomycete *P. ostreatus* with fluorescent probes (Fig. 2). This method clearly visualized the difference in the position of α-1,3-glucan and β-glucan between ascomycete *Aspergillus* and the basidiomycete *P. ostreatus*. In some ascomycete pathogenic fungi, α-1,3-glucans play a key role in avoiding the host immune system mainly by covering the entire mycelium (Yoshimi et al. 2022); however, this seems to not be the case in agaricomycetes such as *S. commune* and *P. ostreatus* and their hyphae are covered by β-glucan*.* It will be interesting to elucidate the function of β-glucan on the surface of vegetative mycelia to understand the evolutionary differences of each cell wall polysaccharide’s function among filamentous fungi.

In agaricomycetes, the structural role of the cell wall is also critical in the formation of fruiting bodies that can withstand the environment, disperse spores, and are flexible enough to change their shape and size during development (Nagy et al. 2023). Because of the beneficial properties of β-glucan from fruiting bodies for human health, only β-glucan’s structure and its functional properties have been focused on (Vetter 2023). In *P. ostreatus*, the cell wall structure of the fruiting bodies was thoroughly analyzed (Table 3)*.* The characteristic cell wall components in the fruiting body of *P. ostreatus* are linear β-1,6-glucan and linear α-1,4-glucan (Palacios et al. 2012; Baeva et al. 2019).

**Table 3:** Structure of different polysaccharides from the *P. ostreatus* fruiting body

Type of linkage	Constitutive monosaccharide	Reference
Glucans		(Reddy Shetty et al. 2021)
α(1→3)	Glucose	(Reddy Shetty et al. 2021)
α(1→4)	Glucose	(Palacios et al. 2012; Reddy Shetty et al. 2021)
α(1→6)	Glucose	(Baeva et al. 2019)
α(1→3),(1→6)	Galactose	(Palacios et al. 2012)
β(1→3),(1→6)	Glucose	(Palacios et al. 2012; Baeva et al. 2019)
Chitin		
β(1→4)	N-acetyl-D-glucosamine	(Baeva et al. 2019)

*P. ostreatus* is one of the few agaricomycetes whose cell walls have been analyzed in both vegetative mycelia and fruiting bodies. Further analysis of the transition of the cell wall from the mycelium to the fruiting body and the details of each cell wall structure will provide a model case for the analysis of cell walls and for understanding the connections between the life cycle and cell walls in basidiomycetes.

### Glucan and chitin synthases

Fungal cell walls are thought to be made by synthases for each component, which are then regulated, bonded to each other, or destroyed by specific enzymes (Brown et al. 2019; Roncero and Vázquez de Aldana 2019; Wagener et al. 2020). Understanding the synthesis of the cell wall is critical for understanding its evolutionary history and differences in structure between phyla, as well as for improving the properties of sustainable materials (see below).

As far as we know, there are no reports with detailed analysis of the mechanism for β-glucan synthesis in basidiomycetes. In ascomycete fungi*,* β-glucans are usually synthesized by β-glucan synthases (FKS), which are transmembrane proteins that convert UDP-glucose into linear β-1,3-glucan chains that are extruded into the cell wall where they bind to various other cell wall components (Gow et al. 2017). Genome sequence information usually predicts a single *fks* gene in ascomycetes, except in yeasts and *Botrytis cinerea*, which have 2–4 to putative *fks* genes. In contrast, many basidiomycetes have multiple putative *fks* genes. In particular, agaricomycetes usually have two *fks* genes (Table 4) (Nagy et al. 2023)*.* The basidiomycete yeast *Cryptococcus neoformans* is an exception, with a single *fks* gene that is essential for both growth and viability (Thompson et al. 1999). *P. ostreatus* has two *fks* genes, *fks1* and *fks2*, and single-gene knockout strains of *fks1* or *fks2* have been successfully obtained (unpublished data)*.* Morphology of Δ*fks1* and Δ*fks2* strains is very different from each other. Variations in the expression patterns of these two genes during different developmental stages have been reported (Nesma et al. 2023). These findings suggest that *fks1* and *fks2* may compensate for each other while playing distinct roles in *P. ostreatus*. Further analysis of these two deletion strains will contribute to our understanding of why many filamentous basidiomycetes have multiple *fks* genes, whereas filamentous ascomycetes have only one.

**Table 4:** Number of predicted genes encoding cell wall synthases

Type of fungi	Species	*chs*	*fks*	*ags*
Basidiomycete (Y/F)^1^	*Cryptococcus neoformans* var. *neoformans* JEC21	8	1	1
	*Ustilago maydis* 521	8	1	0
Basidiomycete (filamentous)	*Lentinula edodes* B17	9	2	3
	*Pleurotus ostreatus* PC9	9	2	1
	*Schizophyllum commune* H4-8	8	2	2
	*Coprinopsis cinerea*	9	4	2
Ascomycete (yeast)	*Saccharomyces cerevisiae* S288C	3	3	0
	*Schizosaccharomyces pombe*	2	4	5
Ascomycete (filamentous)	*Neurospora crassa* OR74A v2.0	8	1	2
	*Aspergillus fumigatus*	8	1	3
	*Botrytis cinerea* v1.0	9	2	1
	*Cochliobolus heterostrophu*s C5 v3.0	9	1	1
	*Cordyceps militaris* CM01	7	1	0
	*Trichoderma reesei* v2.0	8	1	0
	*Morchella importuna*	8	1	0

^1^Life cycle contains both yeast (single cell) and filamentous (multicellular) stages

When it comes to α-glucans, it was reported that, in *C. neoformans*, α-1,3-glucan is synthesized by an α-glucan synthase (AGS) and is not essential for fungal viability, although it has impacts on capsule formation and virulence (Thompson et al. 1999). The number of predicted AGS genes varies largely among fungal species (between zero and five copies) and their phylogeny and evolutionary history are poorly understood (Gow et al. 2017; Nagy et al. 2023). To date, there have been no studies on AGS in agaricomycetes. In *P. ostreatus*, there is only one predicted gene for AGS, but the Δ*ags* strain was viable, suggesting that it is not essential for this fungus (Kawauchi et al., to be published elsewhere). Considering the proposed role of α-1,3-glucans for connecting other polysaccharides in fungal cell walls, it is notable that the single *ags* gene is dispensable in this fungus. By analyzing the detailed cell wall component structure and phenotypes of this strain, the possible roles of α-1,3-glucans in agaricomycetes can be elucidated, and the differences among fungi can be better understood.

Chitin synthases (CHS) belong to the GT2 family of processive polymerizing glycosyltransferases that polymerize UDP-N-acetyl-glucosamine into UDP and N-acetylglucosamine, which are extruded through the cell membrane and linked together to form hydrophobic chitin chains in fungi (Liu et al. 2017; Rogg et al. 2012; Zhou et al. 2019). Analysis of functional roles indicated that different CHSs within an organism perform unique functions that vary between species. For example, in the plant pathogenic yeast-like basidiomycete *Ustilago maydis*, *chs5* and *chs7* shape the yeast cells and hyphae, whereas *chs6*, *chs7*, and *chs1* are involved in virulence and plant infection (Weber et al. 2006). In *A. fumigatus*, on the other hand, some CHSs have weak morphological impacts, while others have no impact on chitin synthesis but still have some impact on vegetative growth (Muszkieta et al. 2014). CHSs are diverse, and it is likely that different CHSs form chitins of different lengths, types, and formations, impacting a large number of other functions across various fungi (Liu et al. 2017). The number of CHSs also varies across species, with most basidiomycetes having 8–9 and ascomycetes 3–8 (Table 4). Phylogenetic analysis suggests the existence of novel basidiomycete-specific clades of CHSs and that the roles of CHSs within these clades may reflect their phylogenetic classification (Schiphof et al., to be published elsewhere). This may be the key to unlocking the reasons behind the structural differences in the cell wall across different phyla. *P. ostreatus* has nine CHSs, with five CHSs located within previously identified phylogenetic classes and four basidiomycete-specific CHSs. Given the structural importance of chitin and the differences in the amount of chitin between ascomycetes and basidiomycetes, basidiomycete-specific CHSs are promising candidates for understanding the differences in cell wall structures and functions of chitin in their life cycle.

## Hydrophobins

**Fig. 3:**
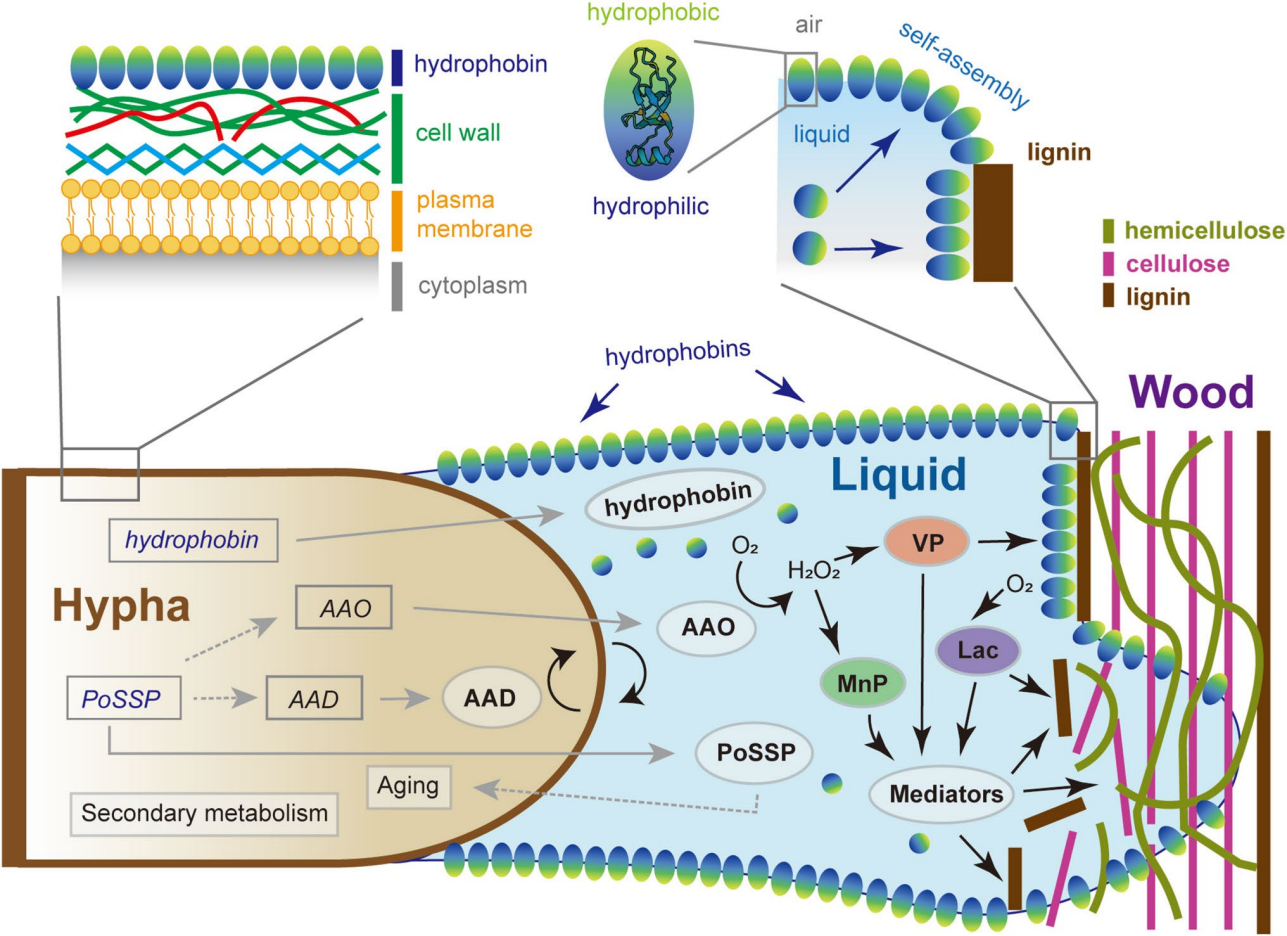
Schematic presentation of the putative roles of hydrophobins in *P. ostreatus* during wood degradation. Hydrophobins self-assemble at the interface between liquid and air, as well as on the hydrophobic surface of wood substrate, and may make a water-filled channel between hyphae and wood surfaces. Aryl-alcohol oxidases (AAO) supply ligninolytic peroxidases including versatile peroxidase (VP) and manganese peroxidase (MnP) with H_2_O_2_ and these peroxidases together with laccase (Lac) attack lignin in wood cell walls either directly or through mediators. Solid grey arrows indicate secretion of proteins; dashed grey arrows indicate indirect effect. The dark brown bars, pink bars, and dark green curved lines indicate lignin, cellulose, and hemicellulose in the structure of wood cell walls, respectively

Hydrophobins have both hydrophobic and hydrophilic parts in their protein structure (Fig. 3) and are typically divided into two classes (class I and class II) based on their hydrophobicity and solubility. Class II hydrophobins are unstable and soluble in sodium dodecyl sulfate (SDS), whereas class I hydrophobins are insoluble in SDS and are the only class of hydrophobins found in basidiomycetes (Wösten 2001). Hydrophobins were first identified in the basidiomycete *S. commune*; these were SC3 from a monokaryon, which is important for aerial hyphae formation, and SC4 from a dikaryon, which is important for fruiting body development (Wessels et al. 1991a; b). The function of SC3 was further studied by making a disruption strain (Δ*SC3*) (Wösten et al. 1994) and was found to help hyphae to break through the water surface into the air by reducing the surface tension (Wösten 2001). SC4 helps maintain gas channels within fruiting bodies by forming a hydrophobic membrane at the surface (Wösten et al. 1999). In other agaricomycetes, hydrophobins play essential roles in fruiting body formation. In *Flammulina filiformi*s, gene silencing of the hydrophobin *hyd9* has obvious effects on primordia development (Tao et al. 2019). Furthermore, in *G. lucidum*, it was reported that the hydrophobin Hyd1 is important for nitrogen regulation and resistance to heat, cell wall, and salt stresses (Qiao et al. 2023). Based on these studies, hydrophobins are assumed to play important roles in the development of vegetative hyphae and fruiting bodies in many basidiomycetes.

In *P. ostreatus*, the first hydrophobin identified was POH1, which was purified from the fruiting body*.* POH2 and POH3 were also identified and purified from the aerial hyphae (Wösten 2001). Fruiting body hydrophobin 1 (FBH1) was also purified from the fruiting body of *P. ostreatus* var. *florida* (N001) (Wösten 2001). Subsequently, three vegetative mycelium hydrophobins (Vmhs) were purified from the N001 strain (Peñas et al. 2002). Vmh2 has been used in characterization of biophysical function as a model of Class I hydrophobins and the ability to self-assemble into amphiphilic films at a hydrophobic−hydrophilic interface, as well as amyloid fibrils was demonstrated (Gravagnuolo et al. 2016; Pitocchi et al. 2023) In later studies, POH1 or FBH1 knockdown using RNAi was reported to affect primordia and fruiting body formation (Nagy et al. 2023; Xu et al. 2021a, b). From this RNAi experiment and phylogenetic analysis, FBH1 and POH1 were identified as the same proteins in different *P. ostreatus* strains.

In recent bioinformatics and RNA sequencing experiments, over 20 hydrophobin genes were predicted in the *P. ostreatus* PC9 strain (Riley et al. 2014; Xu et al. 2021a). Among these, three genes, *vmh2*, *vmh3*, and *hydph16*, were predominantly expressed in the mycelia when cultivated on BWS medium and rice straw medium (Wu et al. 2021a; Xu et al. 2021a). It has been reported that there are some overlapping and specific functions of Vmh2 and Vmh3, as both *vmh2* and *vmh3* are essential for maintaining total mycelial surface hydrophobicity, whereas only *vmh3* affects stress resistance (Han et al. 2023a). Furthermore, gene disruption analyses suggested that the physiological functions of Hydph16 in vegetative mycelia differ from those of Vmh2 and Vmh3 in aerial hyphae formation. Disruption of *hydph16* did not affect hyphal hydrophobicity; however, defects in the hyphal morphology were observed (unpublished data).

Among all previously sequenced agaricomycetes, the number of hydrophobin genes exceeds 10 (Xu et al. 2021a), and *S. commune* and *P. ostreatus* possess 13 and 25 predicted hydrophobin genes, respectively (Table 5). It is noteworthy that, based on the data of 47 species of ascomycetes (Li et al. 2021), the average number of predicted hydrophobin genes was 5.30 and the maximum number was only 13 in these ascomycetes. It remains unclear why there are more hydrophobin genes in basidiomycetes (Rineau et al. 2017), what the functions of each of these genes are, and whether there is functional redundancy. Studies on *P. ostreatus* hydrophobins revealed that the functions of more than 20 predicted hydrophobin genes were partially redundant but tended to have unique functions at different life stages. Moreover, the Δ*vmh3* strains showed a marked delay in lignin degradation on BWS, which was the first demonstration of the possible effect of hydrophobins on natural substrate degradation by a basidiomycete (Han et al. 2023b) (Fig. 3). Combined with their amphipathic physicochemical properties, this finding suggests that basidiomycetous hydrophobins may have diverse functions not only in aerial hyphae and fruiting body development, but also in physiological phenomena at the interface between fungi and the outer environment.

**Table 5:** Number of predicted hydrophobin genes in Agaricomycetes

Species name		Reference
*Pleurotus ostreatus* PC9	25	(Wu et al. 2021a)
*Schizophyllum commune* H4-8	13	(Ohm et al. 2010)
*Coprinopsis cinerea* #326	31	(Muraguchi et al. 2015)
*Flammulina velutipes* KACC43778	10	(Kim et al. 2016a)
*Laccaria bicolor* S238N-H82	13	(Martin et al. 2008)
*Lentinula edodes* W1-26	15	(Chen et al. 2016)
*Lentinus tigrinus*	35	(Wu et al. 2018)
*Agaricus bisporus* H97	22	(Morin et al. 2012)

## Other phenomena

The following phenomena in *P. ostereatus* have been reported to be of interest in understanding the biology of basidiomycetes and agaricomycetes.

Small-secreted proteins (SSPs) are produced by many members of the fungal kingdom and typically contain a signal peptide of less than 300 amino acids (Feldman et al. 2020; Kim et al. 2016b). Despite the abundant production of SSPs (between 40 and 60% of the secretome) in most fungal species, only a few of them have been functionally characterized (Kim et al. 2016b) In *P. ostreatus*, the SSP family (PoSSP) includes genes *ssp1-6*, which are representative examples of well-characterized SSPs (Feldman et al. 2020). Homologs of PoSSPs have been found in 22 fungal species, 16 of which belong to the order Agaricales. The *ssp1* knockdown strains showed reduced expression of genes encoding extracellular aryl alcohol oxidases (AAOs) and intracellular aryl alcohol dehydrogenases (AADs), and overexpression of *ssp1* resulted in elevated expression of genes encoding AAOs and AADs (Feldman et al. 2017). However, the mechanisms underlying the effects of SSP1 on these physiological processes remain unclear (Fig. 3, dashed line). In addition, downregulation of other PoSSP genes through RNAi experiments showed limited lignin degradation on natural substrates but an increase in hemicellulose-degrading capacity (Yarden et al. 2023). Based on these results, the functions of PoSSPs appear to be specialized for the saprotrophic lifestyle of *P. ostreatus*. Another example of an SSP found in *Aspergillus oryzae* is the hydrophobic surface-binding protein, HsbA. HsbA functions similarly to the hydrophobin RolA in the degradation of hydrophobic polymers (Feldman et al. 2020). Phylogenetic analysis revealed three *hsb* homolog genes (*hsb1-3*) in *P. ostreatus*. However, a single disruption of any of the *hsb* genes in *P. ostreatus* had no effect on the morphological phenotype or lignin degradation (unpublished data)*.* Other examples of SSPs include cerato-platanin, a cysteine-rich SSPs that plays an important role in the interactions between fungi and other organisms (Feldman et al. 2020). Based on RNA sequencing data under substrate degradation conditions (Wu et al. 2021a), seven cerato-platanin-coding genes were retrieved from the genome of *P. ostreatus* PC9, and one gene showed predominantly high expression. The function of the gene has been investigated through gene knockout, and thus far, no relationship with morphological formation or lignin degradation has been found (unpublished data). Because of the range of roles and high number of conserved SSP-encoding genes, it is difficult to predict SSP functions in basidiomycetes using homology comparisons and gene expression analysis. Continuous investigations through gene deletion and phenotypic analysis will be helpful in improving our knowledge and elucidating the functions of other SSPs.

In recent years, *P. ostreatus* has been used to study fungal-nematode interactions. Plant parasitic nematodes (PPNs) pose a problem in sustainable agriculture (Al-Ani et al. 2022). Therefore, biological control methods for PPNs are needed to ensure the stable production of agricultural products, and the use of nematophagous fungi is expected to be an effective way to control PPNs. Nematophagous fungi can be divided into four types: (A) nematode-trapping, (B) endoparasitic, (C) nematode-toxin-producing, and (D) egg-parasitic fungi (Jiang et al. 2017). *P. ostreatus* has been recognized as a nematode toxin-producing fungus, and a nematode toxin was recently identified in this fungus (Lee et al. 2023). In this study, mutants lacking the lollipop-shaped structure (toxocysts) were induced by ultraviolet or ethyl methanesulfonate treatment, and these mutants exhibited no cytotoxic activity against the nematode *Caenorhabditis elegana*. Gas chromatography-mass spectrometry (GC–MS) analysis of the volatiles in toxocysts and bioactivity assays of an identified volatile revealed that 3-Octanone is the cause of nematocidal activity in *P. ostreatus*. This study clearly demonstrated how nematode toxin-producing fungi kill nematodes and showed that *P. ostreatus* is a useful model organism for understanding nematode-fungus interactions.

Lectins exist in a wide range of living organisms including fungi and recognize carbohydrates in a non-catalytic, specific, and reversible manner (Singh et al. 2020). In fungi, mushrooms are a rich source of lectin (Singh et al. 2020). Several lectins from mushrooms have been reported to have antiviral, antimicrobial, and immunomodulatory activity (Singh et al. 2020). In the genome of *P. ostreatus* PC9 strain, a total of 24 genes encoding lectins were predicted (Liu et al. 2023). A lectin named POL (*Pleurotus ostreatus* Lectin) was identified from fruiting bodies by multiple independent groups (Perduca et al. 2020). It was reported that POL has medical properties such as food-intake suppression and antitumoral activity (Perduca et al. 2020). The immunogenic activity of POL was also reported in hepatitis B virus vaccine therapy (Gao et al. 2013). In recent protein-structure analysis, POL exhibits a novel fold made up of two homologous β-jellyroll domains which each possess a calcium-dependent carbohydrate-binding site (Perduca et al. 2020). Although the structure and medical proprieties of POL have been analyzed, its physiological role in *P. ostreatus* is still unknown. On the other hand, functions of a galectin-like lectin named Polec2 has been reported recently (Liu et al. 2023). Gene expression of *polec2* was induced in response to attack by the storage mite *Tyrophagus putrescentiae* (Li et al. 2022). The *polec2* overexpression strains show higher resistance against *T. putrescentiae* attack than the wild type. This could be due to induction of more reactive oxygen species, activation of genes involved in mitogen-activated protein kinase (MAPK) pathways, or changes to jasmone and salicylic acid production (Liu et al. 2023). As physiological functions of many lectins in mushrooms are still unknown, results of the Polec2 study highlight the usefulness of molecular genetics of *P. ostreatus* in understandings functions of mushroom lectins.

## Conclusions

With the recent progress in molecular genetic approaches, including post-genomics and genome editing, *P. ostreatus* provides a good experimental platform for investigating the molecular mechanisms underlying various physiological phenomena in mushrooms. Genome analysis has revealed that many agaricomycetes have redundant genes for substrate degradation, complex and multilayered signal transduction systems, and many genes encoding proteins with unknown functions. Comparative transcriptomics have demonstrated that the expression of many genes is altered under various conditions. It is sometimes difficult to understand the core mechanisms that lead to fundamental changes in cells and side effects. Along with forward genetic analysis, the knockout of multiple genes may help elucidate the molecular mechanisms underlying these complex phenomena. By comparing with other mushrooms, common principal mechanisms among many mushrooms as well as species-specific unique mechanisms will be uncovered using *P. ostreatus* as a model.
